# The Relationship between Quality of Life with Marital Satisfaction in Nurses in Social Security Hospital in Zahedan

**DOI:** 10.5539/gjhs.v8n2p178

**Published:** 2015-06-25

**Authors:** Maliheh Gharibi, Gholamreza Sanagouymoharer, Fariba Yaghoubinia

**Affiliations:** 1 Faculty member, Department of nursing, Islamic Azad university of Iran, Zahedan branch, Zahedan, Iran; 2 Faculty member, Department of psychology, Islamic Azad university of Iran, Zahedan branch, Zahedan, Iran; 3 Pregnancy Health Research Center, Zahedan University of Medical Sciences, Zahedan, Iran

**Keywords:** quality of life, marital satisfaction, nurses

## Abstract

**Background:**

Marital satisfaction is one of the most important determinative factors of healthy function in family and can be affected by some factors.

**Aim:**

This study was conducted aimed to determine the relationship between quality of life and marital satisfaction in nurses in Social Security hospital in Zahedan.

**Method:**

In this descriptive and correlational study, the population was the all of the nurses in various wards in Social Security hospital in Zahedan. The sample size was 103 and data collection was done through quality of life questionnaire (War and Sherborn) and Enrich Marital Satisfaction Scale. Data analysis was done through SPSS15 and using pearsons’ correlation coefficient and stepwise regression.

**Results:**

The aspects of physical functioning, role limitations due to physical health problems, role limitation due to emotional problems had a significant positive correlation and the bodily pain had a significant reverse correlation with aspects of marital satisfaction. The aspects of role limitations due to physical health problems and bodily pain were predictors of marital satisfaction.

**Conclusion:**

The results of study demonstrated the importance of pay attention to family issues and marital satisfaction and in this regard, the promotion of all aspects of quality of life is essential.

## 1. Background

Work is an important part of everyone’s life. Satisfaction with work is an important part of everyone’s life as it can affect a number of basic human needs such as nurturing the mind and body, social relationship, creation of sense of worth, self-confidence and competency. Also; it may be the main source of dissatisfaction (Peiman Pak et al., 2012).

The entrance of people to work world due to nature of some jobs such as nursing has caused the confrontation of them with some phenomena named workplace conflict, family conflict and work–family conflict. In these conflicts, Job commitments and family commitments interact with each other and affect the quality of life of people; and consequently lead to occurrence of problems such as marital dissatisfaction (Rajabi et al., 2013).

Successful marriage causes satisfy the physical and psychological needs in people and in the cases of unsuccessful marriage, the couples and their children encounter with severe psychological trauma. Therefore; the survey of marital satisfaction and the factors that can affect of durability of marital life, is very important (Padash et al., 2012).

Marital satisfaction is one of the most important determinative factors of healthy function in family (Greef et al., 2000). Kaplan and Maddux (2002) stated that the marital satisfaction is a personal experience in marriage and it can be assessed only by the couples. This occurs through their response to satisfaction rate from marital relationship. They believed that the marital satisfaction is dependent to individuals’ expectations and beliefs (Kaplan & Maddux, 2002).

Marital satisfaction consisted of four domains such as physical and sex appeal, understanding, attitude and investing (Mobarak Abadi et al., 2014).

The mentioned satisfaction was not easily accessed (Myers, 2004). From the beginning of marriage, differences and disagreements between man and woman in majority issues and unsolved problems can face them with threats in marital satisfaction and stability (Tallman & Hsiao, 2004).

In Fizer’ study (2002), results demonstrated that some factors have critical role in marital satisfaction such as gladness in relation to self and life, positive self-esteem, self-contact, sense of autonomy, real expectations, proper interpersonal skills, sense of responsibility for others and having a positive attitude in difficult living conditions. Overall, quality of life increases marital satisfaction and mental health (Kazemi et al., 2011).

Quality of life is a sense of well being that is arises from the satisfaction or dissatisfaction in various important aspects of life (Sammarco, 2001). For each person, quality of life is dependent on his unique perception from life and life satisfaction; in spite of the relationship with family, friends and community. In addition, the person is satisfied from psychological, social, economical, cultural, religious and sexual aspects (Cella, 1994).

The studies show that the numerous work pressures in nurses had negative consequences in their family and social issues and had caused disruption in their common social activities with their spouse. This led to increase the interpersonal conflicts and affect their quality of life and satisfaction (Elquist, 2004).

According to the importance of balance performance in family and prevention of its disconnection, the recognition and assess the related factors with marital satisfaction which is the foundation for a strong family, is very important. Therefore, with attention to effective factors on marital satisfaction, it is expected that with the increase of level of marital satisfaction, many psychological, emotional and social problems would be decreased in family and in the community. Also; with the improvement of the marital satisfaction level and Life satisfaction, the people carry out their duties with greater confidence and peace of mind (Heidari & Eghbal, 2010)

Due to the unique nature of nursing profession and high stress conditions for nurses, the specific problems have been created for them and with regard to the problems in nurses’ health status due to satisfaction, it is important to survey the marital satisfaction of this stratum of society. Marriage life has the various aspects such as financial issues and emotional relationship. Due to the increasing number of family roles, individuals’ duties and the challenge concerning to the balancing between job and family roles, this study intent to response to this question “Does the nurses’ quality of life affect on their marital satisfaction?”

Limited research have been examined the relationship between quality of life and marital satisfaction. Vibha, Saddichha, and Akhtar (2010) compared the quality of life and marital adjustment in patients with epilepsy and psychiatric patients. 60 patients (30 Epileptics and 30 bipolar patients) were studied. Results showed that there is no significant relationship between quality of life and marital adjustment in two groups (Vibha et al., 2010).

The results of Pereira et al study (2011) in which quality of life, behavioral issues and marital adjustment were examined, also demonstrated that there is significant positive relationship between high level of quality of life and marital adjustment. Also; the findings of Gameiro et al. (2011) showed that there is significant positive correlation between marital satisfaction and quality of life and the promotion of quality of life causes the increase in all aspects of marital satisfaction (Pereira et al., 2011; Gameiro et al., 2011).

Whisman et al in study on 744 American couples confirmed the existence of relationship between anxiety and depression with marital satisfaction. In this study, they realized the relationship between psychological health status and couples’ satisfaction (Whisman et al., 2004).

The study by Rostami et al. (2013) was conducted to determine the effectiveness of quality of life based treatment on psychological well-being in incompatible couples. Results demonstrated that this treatment causes the improvement of psychological well-being and components of environmental skills, personal growth, positive relationship with others and self-acceptance (Rostami et al., 2013). The improving of quality of life causes the increase of marital satisfaction and psychological well-being.

The study by Banaian et al. (2006) titled “the relationship between psychological health and marital satisfaction in married women also showed that there is significant relationship between marital satisfaction and psychological health in women and the women with high psychological well-being have better marital satisfaction. Depressed mood, loss of energy, a sense of lack of pleasure in life activities and consequently; decline in individual and social function in marriage were manifested as relational and marriage problems (Banaian et al., 2006).

Also, the results of Alavian et al study (2006) showed that the subscales of role limitations due to physical health problems, bodily pain, social functioning, mental health, vitality, mental and physical health from quality of life questionnaire have the significant positive relationship with the overall score of marital adjustment, understanding, express feelings toward wife and marital satisfaction. Role limitation due to emotional problems and perception of general health has correlation with overall score of marital adjustment, express feelings toward wife and marital satisfaction. Physical functioning has no correlation with none of the subscales of quality of marital relationship (Alavian et al., 2006).

According to above mentioned, the current study was conducted aimed to determine the relationship between quality of life and marital satisfaction in nurses in Social Security hospital in Zahedan.

## 2. Method

In this descriptive-analytical research, study population was the all of the nurses in various wards in Social Security hospital in Zahedan. The inclusion criteria were married and had at least one year work experience. All of them were selected as study sample and 103 nurses were studies. After entering the subjects to study, questionnaires gave them in workplace and they were asked for completion of questionnaires in workplace. Response rate was 100 percent.

Research tools including two questionnaires: Quality of work life questionnaire (SF-36) which was used for clinical work, evaluation of health Policy and studies related to general population. The 36-items form of this questionnaire was designed by War and Sherborn in 1992 in America. Its validity and reliability were examined in various groups. In this questionnaire, the assessment of physical and psychological health was done through the combination of eight domains of health.

Physical functioning including questions 3-12; public health (1, 33-36), limitations due to physical health problems (13-16), role limitation due to emotional problems (17-19), bodily pain (21, 22), social functioning (20, 32), vitality (23, 27, 29, 31) and mental health (24-26, 28, 30) that measure the quality of work life. The item 2 is not included in any of the subscales and only is added to total score of questionnaire. The lowest score is 0 and the highest is 100.

In study by Farhadi et al (2011), the obtained correlation coefficient was in range of 0.45-0.72. The alpha cronbach was calculated 0.8 for the whole of the questionnaire (Farhadi et al., 2011). Motamed et al. (2002) in Iran calculated the alpha cronbach 0.87 for this questionnaire (Motamed et al., 2002).

### 2.1 Enrich Marital Satisfaction Scale

In current study, Enrich Marital Satisfaction Scale was used. This questionnaire was designed by Olson & Fournier & Druckman (1992) in order to evaluate the potentially problematic context and identify the context of power and improvement the marital relationship. This questionnaire has two forms of 115 questions and 125 questions including 12 subscales. Its subscales including Idealistic Distortion, Marital Satisfaction, Personality Issues, marital Communication, Conflict Resolution, Financial Management, Leisure Activities, Sexual Relationship, Children and Parenting, Family and Friends, Equalitarian Roles, and Religious Orientation. Because of the large number of the questions in the original form, it causes fatigue in subjects. The Iranian version of Enrich Marital Satisfaction Scale was validated in Iran. Soleimanian (1994) prepared the short form of this questionnaire with 47 questions (Pourghaffari, Pasha, & Attari, 2009).

The short form of this questionnaire was used in current study in which 11 factors including marital satisfaction, personality issues, marital relationship, conflict resolution, financial supervision, leisure time activities, sexual relationship, children and parenting, relatives and friends, roles of gender equality and religious orientation. This questionnaire uses 5 point likert scale. Each item has 1-5 score and scoring of some of them is reverse.

Amiri (2010) quoted Vadesbay who conducted extensive research concerning the validity and reliability of this questionnaire, as; its reliability with test-retest and discrimination power was reported 0.65-0.94 and 0.9 respectively. (Amiri Majd et al., 2010). Data analysis was done through SPSS 15 and using Pearson’s correlation coefficient and stepwise regression.

## 3. Results

The mean age of nurses was 32.8±4.6. The majority of nurses were women (81.6 percent). 35 percent of subjects had the marriage duration between 6-10 years and 11 percent had the marriage duration more than 15 years.

Among the eight aspects of nurses’ quality of life, the highest score was related to physical function and the lowest score was related to physical problems (Table 1).

**Table 1:** The mean and standard deviation of aspects of quality of life in nurses

Aspects of quality of life	Mean	SD
Physical functioning	64.6	28.1
Role limitations due to physical problems	51.4	37.9
Bodily pain	53.9	24.1
General health perception	57.5	20.8
Vitality	57.2	20
Social functioning	61.4	23.8
Role limitations due to emotional problems	54	42
Mental health	63.2	18.4

*=p<0.05; **=p<0.01.

Table 2 shows the correlation coefficient between various aspects of quality of life and marital satisfaction. The results in this table show the significant correlation in two levels of significances.

**Table 2:** The correlation of various aspects of quality of life and marital satisfaction

	Physical function	Public health	role limitations due to physical health problems	role limitation due to emotional problems	Bodily pain	Social function	vitality	Mental health
Marital satisfaction	0.3	0.02	0.33	0.34	-0.41	0.00	0.01	0.00
Personality issues	0.38	-0.02	0.4	0.25	0.33	0.13	0.14	0.04
Conflict Resolution	0.3	-0.03	0.34	0.37	0.44	0.00	0.09	-0.05
Marital relationship	0.2	-0.12	0.26	0.27	-0.23	0.06	0.07	-0.03
Financial Management	0.47	-0.05	0.46	0.37	-0.4	-0.07	0.11	0.03
Leisure Activities	0.37	0.08	0.3	0.25	-0.2	0.07	0.02	0.00
Sexual Relationship	0.01	-0.00	0.14	0.22	-0.23	0.15	-0.07	0.00
Children and Parenting	0.22	0.04	0.28	0.33	-0.22	-0.05	0.06	0.06
Family and Friends	0.21	0.00	0.27	0.27	-0.23	0.09	-0.07	-0.01
Equalitarian Roles	0.34	-0.04	0.32	0.32	-0.34	-0.02	0.07	-0.02
Religious Orientation	0.35	0.04	0.3	0.22	-0.11	0.06	-0.02	-0.07
Overall score of marital satisfaction	0.38	-0.01	0.41	0.39	-0.39	0.04	0.04	-0.00

According to Table 3, stepwise regression was used for prediction of the total score of marital satisfaction with attention to aspects of quality of life. At first the role limitations due to physical health problems and then; the bodily pain entered the regression equation. Results showed that these two aspects predicted 1.7 and 2.1 percent of total variance of marital satisfaction respectively. Other aspects of quality of life don’t have the criteria for entering the equation and were deleted from equation. The aspect of role limitations due to physical health problems has significant positive relationship (Beta=0.283, p<0.000) and bodily pain has significant negative relationship (Beta= -0.249, p=0.01) with total score of marital satisfaction. The aspect of role limitations due to physical health problems was the positive predictor and bodily pain was the negative predictor of marital satisfaction.

**Table 3:** The stepwise regression of overall score of marital satisfaction based on aspects of quality of life

Variable	R	Change	B	Std. Error	Beta	T	sig
role limitations due to physical health problems	0.41	0.17	4.780	1.756	0.283	2.722	0.00
Bodily pain	0.46	0.04	-2.651	1.107	-0.249	-2.395	0.01

## 4. Discussion

The current study was done aimed to investigate the relationship between quality of life and marital satisfaction in nurses in social security hospital in Zahedan. Results demonstrated that the aspects of physical function, role limitations due to physical health problems and role limitation due to emotional problems has significant positive relationship and bodily pain has significant reverse relationship with aspects of marital satisfaction. Also, there is a significant positive relationship between total score of quality of life and marital satisfaction (p=0.01). The aspect of role limitations due to physical health problems and bodily pain were the predictors of marital satisfaction.

These finding are consistent with other studies in which there is a significant relationship between marital satisfaction and quality of life (Pereira et al., 2011; Gameiro et al., 2011; Rostami et al., 2013; Ghaffari et al., 2013; Alavian et al., 2013), marital satisfaction and mental health (Whisman et al., 2004; Alipour et al., 2013, Banaian et al., 2006), balancing responsibilities and couple cooperation (Kijer& Riely, 2000), work and life conflict and life satisfaction (Chiu, 1998) and psychotherapy based on quality of life (Padash et al., 2012). These studies showed that the conflicts of work and life have negative effects on marital satisfaction. Also, life satisfaction has positive effect on marital satisfaction. There is significant relationship between mental health as an indicator of quality of life and marital satisfaction, also; mental health can predict the marital satisfaction. Only in Vibha et al study (2010), the relationship between quality of life and marital adjustment was not reported.

The stressful job situations and their outcomes lead to incompatibility and stress in relationship between couples. Work and life are the two related domains in which problems and issues of each of them can affect on another. In other words; the existence of tension and stress in each of these two domains causes the problems in role playing and leads to discomfort and dissatisfaction between couples (Peiman Pak et al., 2012).

The results of current study showed that the role limitation due to physical health problems has significant effect on marital satisfaction. Because of most of the time of nurses spend in the workplace and their responsibilities are enormous, perhaps they face limitations in their responsibilities and roles especially in the home environment. As a consequence, they may have some problems in their wife roles and this is a source of marital dissatisfaction.

Also, there is a significant reverse relationship between bodily pain and marital satisfaction. It can be said that the physical problems and decline in physical health in women decrease the chance of satisfactory relationship and consequently; causes the reducing the relationship with others especially with spouse and actively express of love and emotions. Also, because of the high workload of nurses, they are susceptible to involvement in physical and psychological diseases and this lead to marital dissatisfaction.

As expected, when an individual’s job interferes with other responsibilities, the sense of role conflict becomes more and marital satisfaction becomes less (Rajabi et al., 2013). In other words, with decline in individual’s health, not only her relationship changes with her husband, but also the behaviors of her spouse are affected. In fact, the disease is the stressful situations for both the parties (Alavian et al., 2006).

Based on our results, the all aspects of quality of life in nurses should be considered more. Implementing the in-service training classes and related workshops and attention of hospitals managers to these aspects is essential. In this regard, promote and increase the marital satisfaction of nurses and increase their knowledge in this area is necessary.

The lack of reliable methods of clinical evaluation such as interview is one of the limitations in current study. The research population was the nurses in social security hospital in Zahedan, thus the generalizing the results to all of the nurses in another limitation.

Also, there is a possibility of bias in measurement of quality of life. As in the Mazaheri’s study (2010) titled “the survey of factors affecting the bias in the measurement of quality of life” results showed that there is a negative skewness in all of the scales in distribution of measured variables. The type and format of scale are the important and effective factors in measuring the quality of life (Mazaheri, 2010). Also, the implementing of educational workshops for nursing staff and nursing students in order to promotion of quality of life should be done.

## 5. Conclusion

The results of current study showed the necessity of pay attention of health issues planners, psychologists and psychiatrists to family issues and marital satisfaction, because of the family is the most fundamental social institution and its health or disease has deep effect on the various aspects of social life, the health of family lead to health, peace and stability in community.
